# Serum intact fibroblast growth factor 23 in healthy paediatric population

**DOI:** 10.1515/med-2021-0288

**Published:** 2021-07-06

**Authors:** Malgorzata Stanczyk, Slawomir Chrul, Krystyna Wyka, Marcin Tkaczyk

**Affiliations:** Department of Pediatrics, Immunology and Nephrology, Polish Mother’s Memorial Hospital Research Institute, Rzgowska 281/289, Lodz 93-338, Poland; Department of Pediatrics, Oncology and Hematology, Medical University of Lodz, Lodz, Poland; Department of Pediatrics, Preventive Cardiology and Immunology of Developmental Age, Medical University of Lodz, Poland; Department of Pediatrics, Immunology and Nephrology, Polish Mother’s Memorial Hospital Research Institute, Lodz 93-338, Poland

**Keywords:** child, fibroblast growth factors, renal insufficiency

## Abstract

**Introduction:**

It is believed that fibroblast growth factor 23 (FGF23) can become an early biomarker of chronic kidney disease progression. Data on FGF23 age dependency are inconsistent. We present the results of the cross-sectional study concerning FGF23 levels in healthy Polish children.

**Material and methods:**

This study was conducted in 121 children aged 0–18 years. Kidney function and intact FGF23 levels in serum were assessed. Differences between age groups and according to gender were analysed.

**Results:**

The difference in FGF23 between age groups and according to gender was statistically insignificant. In the youngest and the oldest group, a trend to higher FGF23 levels was observed. FGF23 level in girls tended to be higher than boys, apart from the age group between 1 and 4 years. There was a negative correlation between eGFR and FGF23 (*r* = −0.26, *p* < 0.05) – strong in girls (*r* = −0.38, *p* < 0.05), but not in boys. In each age group, we found no significant correlation between eGFR and FGF23.

**Conclusions:**

Our study supports the evidence that the FGF23 level in paediatric population is not age or sex dependent. The results can serve as a reference point under clinical conditions and for other studies on the topic.

## 1 Introduction

**Figure 1:**
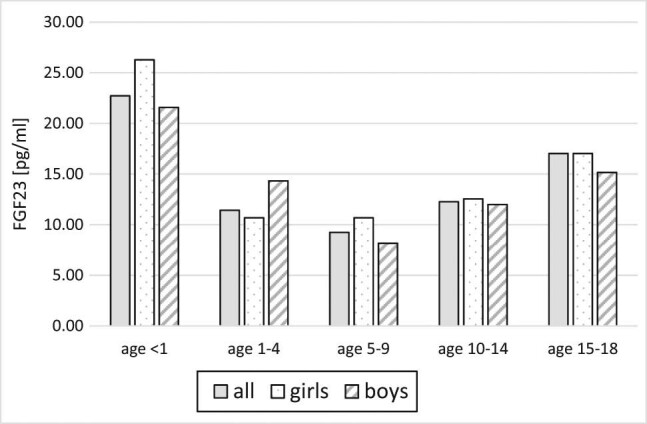
FGF23 levels in age groups – total and with sex distribution. Median of values is presented, *p* > 0.05.

**Figure 2:**
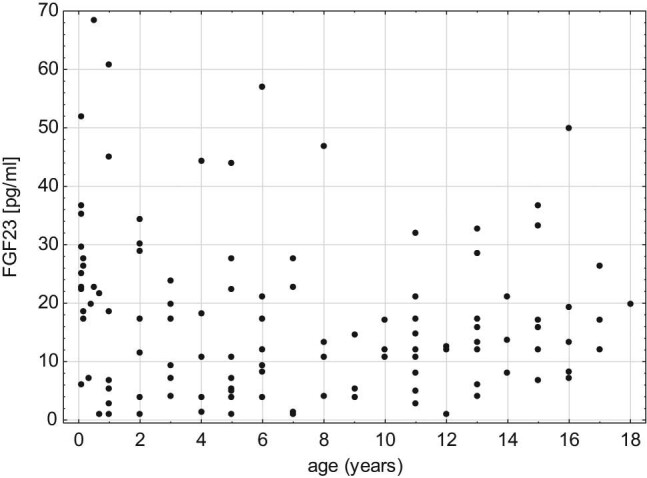
Scatter plot of serum FGF23 depending of the age.

Fibroblast growth factor 23 (FGF23) was described in 2000 as a phosphaturic hormone acting as a regulator of calcium–phosphate balance affecting serum phosphates, parathormone (PTH) and vitamin D (1,25-(OH)_2_-vitamin D3) levels [1,2,3]. Protein FGF23 of molecular weight 32 kDa is synthesised mainly by osteocytes and osteoblasts [4]. FGF23 acts through FGF receptors in cooperation with Klotho protein. Activated receptors suppress the function of two sodium-phosphate co-transporters in proximal convoluted tubules in kidneys (NPT2 and NPT2c) inducing phosphaturia and suppressing the formation of 1.25-dihydroxy-vitamin D by inhibiting renal 1α-hydroxylase activity [4,5]. FGF23 circulates in serum in two forms – intact, active form and inactivated c-terminal fraction (cFGF23) [6]. It is confirmed that in end-stage kidney disease, the FGF23 level in serum increases [7,8,9]. A growing number of clinical observations and experimental research involving animals shows that the FGF23 serum level increases significantly in chronic kidney disease much earlier than previously used biomarkers (creatinine, phosphates, vitamin D and PTH) [10]. Therefore, it is believed that it can become the early and sensitive biomarker of progression of chronic kidney disease (CKD) [11]. In view of physiological age dependency of serum calcium and phosphates, it is worth to investigate if FGF23 is also changing with age, especially since some data suggest such a relation [12]. In this article, we present the results of the cross-sectional study concerning FGF23 levels in the healthy Polish children population (Figures 1 and 2).

## 2 Materials and methods

This study was conducted in children aged 0–18 years randomly selected among children hospitalised on planned reasons in the Department of Pediatrics, Immunology and Nephrology of Polish Mother’s Memorial Hospital Research Institute, Lodz, Poland. The study group comprised of patients with no clinically significant disease entities who were hospitalised in our centre for diagnostic reasons (i.e. diagnostics of recurrent respiratory tract infections, abdominal pain) or minor surgical procedures. Eventually, generally healthy children were enrolled into the study, with negative history of chronic diseases, including chronic kidney disease, heart diseases, gastrointestinal diseases, endocrine disorders and other conditions, which could affect calcium–phosphate balance. To confirm the lack of health conditions that could interfere with the study aims, routine biochemical assessments were performed, and the results referred to reference ranges for age and sex when applicable. All results were within reference ranges. Patients with abnormal results were excluded from the study.

Children with vitamin D deficiency (<30 ng/mL) or after trauma including bone fracture within 3 months before enrolment were excluded. Local Bioethics Committee approved the study protocol. Consent of the caregivers was mandatory, as well as in the case of children aged 14 years and older.

Kidney function was assessed by creatinine levels and the estimated glomerular filtration rate (eGFR) by Schwartz equation (*k* × height [cm]/serum creatinine), with *k* values different for age and sex groups (*k* = 0.450, 0.55, 0.7 for children aged <12 months, <12 years, and girls ≥13 years and boys ≥13 years, respectively).

Intact FGF23 level in serum was assessed with ELISA kits (R&D Systems, Inc., USA) with 3 pg/mL sensitivity. Blood sample for assessment was collected fasting in the process of planned control biochemistry tests. Until assessment, the samples were stored at −80°C, but no longer than 3 months.

Normality of distribution was tested with the Shapiro–Wilk test. Given the nonnormal distribution of FGF23 levels, its values were described by a median with mean deviation. The differences in proportion were tested with the chi-squared test. Pearson’s correlation test and Spearman’s rank test were used to assess proportion of correlation between variables. A significance level of *p* < 0.05 was considered significant.

## 3 Results

This study was conducted in 121 children (M, 66, *p* > 0.05). Sex distribution, body mass index (BMI), kidney function expressed as eGFR (median and interquartile range), and FGF23 levels (median and mean deviation) in age groups are presented in Table 1, and results of biochemical assessments during enrolment to the study are presented in Table 2. Haemoglobin concentration did not differ between boys and girls (13.2 vs 12.8 g/dL respectively, *p* = 0.14).

**Table 1:** Sex distribution, BMI, serum FGF23 level and eGFR in the age groups

Age (years)	Boys	Girls	Total	BMI (kg/m^2^) Median (interquartile range)	FGF23 level (pg/mL) Median (99pc)	eGFR (mL/min/1.73 m^2^) Median (interquartile range)
<1	8	11	19	14.5 (13.6–15.4)	22.73 (68.39)	106.5 (86.62–129.00)
1–4	19	9	28	15.8 (14.9–17.2)	11.44 (56.74)	143.00 (129.25–157.20)
5–9	19	13	32	15.6 (14.8–16.8)	9.25 (53.96)	170.50 (155.37–196.16)
10–14	14	13	27	19.9 (17.0–21.9)	12.27 (32.55)	153.12 (143.50–173.00)
15–18	6	9	15	20.0 (17.6–22.6)	17.03 (48.08)	144.8 (124.9–165.81)
Total	66	55	121			

**Table 2:** Basic biochemical parameters assessed at the enrolment to the study

Parameter	Mean (SD) Median (range)
Serum creatinine (mg/dL)	0.45 (0.19)
	0.4 (0.2–0.9)
Estimated glomerular filtration rate (mL/min/1.73 m^2^)	147 (35)
	146.7 (63.3–212.7)
Haemoglobin (g/dL)	13 (1.5)
	13.1 (10.5–15.8)
White blood cell count (cells/µL)	8,990 (3,600)
	7,000 (4,170–17,250)
C-reactive protein (mg/dL)	<0.5
Alanine aminotransaminase (IU/L)	28 (10)
	31.5 (10–45)
Serum calcium (mmol/L)	2.58 (0.12)
	2.76 (2.48–2.76)
Serum phosphates (mmol/L)	1.77 (0.32)
	1.76 (1.31–1.8)
25-OH vitamin D (ng/mL)	38.3 (30–63.8)
PTH (pg/mL)	30.6 (20.81–43.87)

The difference in FGF23 between age groups was statistically insignificant. 99pc values for different age groups were between 32.55 pg/mL in 10–14 years group and 68.39 pg/mL in the group younger than 1 year.

In the youngest and the oldest group, a trend to higher FGF23 levels was observed but without statistical significance.

Differences between girls and boys were insignificant. FGF23 level in girls tended to be higher than boys, apart from the age group between 1 and 4 years.

According to the kidney function, we have found a negative correlation between eGFR and FGF23 (*r* = −0.26, *p* < 0.05). We have found strong negative correlation between FGF23 and eGFR in girls (*r* = −0.38, *p* < 0.05) but not in boys. In each age group, we found no significant correlation between eGFR and FGF23.

## 4 Discussion

Many medical disorders were described in which oversynthesis of FGF23 results in hypophosphatemia, i.e., X-linked familial rickets, autosomal dominant and recessive forms of hypophosphatemia, and tumour-induced osteomalacia [1,13,14]. Clinical studies revealed a positive correlation between serum FGF23 level and higher left heart ventricle mass with a trend to its hypertrophy, deterioration of coronary arteries structure, and endothelial dysfunction [11,15]. Ix et al. study in the adult population revealed that during the progression of chronic kidney disease, FGF23 levels begin to increase under eGFR of 90 mL/min/1.73 m^2^ [16]. In the paediatric population with CKD assessed by Portale et al., cFGF23 increased already at eGFR of 30–90 mL/min/1.73 m^2^ [9]. In the majority of the CKD participants, higher values of FGF23 were observed before the increase of serum parathyroid hormone and phosphorus. The authors concluded that in children with predialysis CKD, high cFGF23 is the earliest detectable abnormality of mineral metabolism. Therefore, the hypothesis that the serum FGF23 level could outrun first biochemical symptoms of calcium–phosphate imbalance in patients with early stages of chronic kidney disease is justified. Serum FGF23 level could be a promising early marker of calcium–phosphate imbalance giving an opportunity to react by implementing adequate therapy before the first symptom occurs.

Calcium–phosphate balance markers differ according to their age dependency. In the paediatric population, serum 1,25-hydroxylated vitamin D and PTH are age independent. Calcium and phosphate levels drop with age [17]. As FGF23 is a phosphaturic hormone, it could be hypothesised that the lower serum phosphates the higher serum FGF23. In the study by Portale et al. on CKD paediatric patients, it was showed that in patients with eGFR 30–69 mL/min/1.73 m^2^ when the increase of FGF23 is already marked, serum phosphates are the lowest reflecting probably FGF’s23 phosphaturic action [9]. In the later stages of CKD, the overactivity of parathyroids and kidney phosphaturic disability prevail with the increase of phosphataemia [18]. Conversely, considering healthy children, Gkentzi et al. in a study on 159 children aged 2–18 years found a weak positive correlation between serum phosphate and FGF23 [19]. This finding is inconsistent with the study by Mitchell et al. who demonstrated that despite phosphate levels in healthy populations correlate negatively with the age (*r* = −0.49, *p* < 0.001), serum FGF23 levels are not directly associated with phosphates serum and urinary levels [20]. They concluded that FGF23 is not associated with age-related changes in phosphates.

Data on age dependency of FGF23 are incoherent. Mitchell et al. in their analysis of 90 girls aged 9–18 years concluded about lack of correlation between age and FGF23 or c-terminal FGF23 [20]. The authors hypothesised that similar to age-independent PTH, FGF23 rather than being the primary regulator of serum and urinary phosphate excretion acts as a system defending the phosphate set point determined by other factors. Gkentzi et al. also did not observe an association of either form of FGF23 with age [19]. However, Fischer et al. revealed by analysis of 424 healthy children that cFGF23 is significantly age dependent with highest values in early infancy and adolescence ((*r* = −0.22, *p* < 0.01) [12]. Our results did not support such a hypothesis – we did not notice a correlation between FGF23 serum levels and age. We found that FGF23 in healthy paediatric patients does not differ between girls and boys as well. We have noticed only a trend for higher values in girls and the youngest and the oldest group of children, without statistical significance. Fischer et al. observed similar but significant distribution of c-terminal FGF23 as FGF23 in our study – higher levels in infancy and until 3 years of age, stable low levels in childhood, and again increase in those older than 12 years. In contrast to our study in the cited paper, the c-terminal inactive FGF23 was assessed, whereas we assessed active FGF23. This could be suggestive that c-terminal but not intact FGF23 is age dependent. Moreover, in that article, it was stated that cFGF-23 was positively correlated with serum phosphate concentration. We found no such correlation – it could be associated with different phosphate status in assessed children.

In our study the youngest children tended to have higher levels of FGF23. This is also the age group with the highest phosphate values, as the reflection of bone formation. The explanation of this phenomenon seems very interesting and important. One of the FGF23 functions is protection from excessive levels of phosphorus negatively affecting osteoblasts resulting eventually in cell death [2]. Hypothetically higher phosphates induce FGF23 to stimulate phosphate renal excretion and suppress intestinal absorption. The precise mechanism of association among dietary phosphate load, high serum phosphate, and increased synthesis of FGF23 is still not clear [2,21,22]. Physiologically associated with bone formation and growing higher phosphates in infancy and early childhood could be explained by this hypothesis. But it is still unclear why adolescents, despite lower levels of phosphates, tended to have higher serum FGF23. Supposedly, it could be connected with a higher burden of phosphates in the western diet – FGF23 here would be also acting as protection from excessive phosphate load. In the present study, we found no correlation between phosphate levels and FGF23 values – the FGF23 values in infancy and adolescence were not reflected by higher phosphate concentrations in corresponding age groups. However, it needs to be emphasised that FGF23 levels were not different in age groups, and the only trend was marked without significance. Lack of association between FG23 and phosphate levels in the healthy population should not be translated into CKD population as the protective FGF23 action against excessive phosphate burden was found [2].

We found a tendency to higher values of FGF23 in the youngest children and adolescent girls. Such a trend could be hypothetically explained by associations between FGF23 and iron deficiency. Studies performed in adult and paediatric population showed that intact and c-terminal FGF23 is correlated with poor iron status, and a decrease in FGF23 level can be noticed after iron supplementation [23,24,25]. The pathophysiology of this finding still needs clarifying. Infants of mothers with pregnancy iron deficiency and adolescent girls can be considered as iron deficiency risk populations. However, in our study, we did not assess the iron status of patients because they all were healthy without anaemia. In light of the former and FGF23 level, age independency considerations on the association between FGF23 and iron status can be only hypothetical.

In different conditions affecting calcium–-phosphate balance characterised by stimulation of FGF23, its increase is in general very high, way beyond upper range limits for the healthy population. In a small Polish study, in 37 children assessed with chronic kidney disease stage 3, median plasma FGF23 level ranged from 179 to 750 ng/L (1 ng/L = 1 pg/mL), while in the control healthy group, it was 55 ng/L (IQR, 30–108) [8]. In Portale et al. study the median plasma FGF23 level was 2.4 times higher in CKD 2–4 patients than in 42 healthy children of comparable age of 12 ± 4 years (138 RU/mL vs. 57 RU/mL, respectively; according to the manufacturer 1 RU/mL is roughly 2 pg/mL). Individuals with CKD 2 had median FGF23 equal to 93 RU/mL (around 200 pg/mL) without any other changes in calcium–phosphate balance [9]. Noteworthy, in this study, as “healthy” were considered children whose eGFR was >70 mL/min/1.73m^2^. Therefore, the children with mild kidney dysfunction were not excluded, so obtained normal value 0f FGF23 could be overestimated. In 2012, Fischer et al. published paediatric age- and gender-related percentile charts and z-scores for cFGF-23. The 97th percentile of cFGF23 ranged from 324 kRU/L in infants to 100 kRU/L in 18 years old [12].

It is difficult to compare all available data on the subject because of different methodologies concerning the assessed marker – some of studies based on intact FGF and others on c-terminal FGF23. Some data suggest superiority of c-terminal FGF23 assessment because of lower variability of results, the better precision of assessment, and better correlation with worsening of kidney function [26,27]. In our study, intact FGF23 level was assessed so it is impossible to compare the results with the German study. In general, measured levels of cFGF23 are higher than intact FGF23, but there are no data about converting one to another. In our healthy population, we have noticed a small range span of intact FGF in each age group. The 99th percentile for FGF23 range in each group varied from 32.55 pg/mL in children aged 10–14 years to 68.39 pg/mL in children younger than 1 year. As reported by other studies, values under disease conditions are significantly higher than detected in the healthy population of our study [8]. Given the unavailability of serum intact FGF23 reference values in paediatric population, our study provided valuable contribution to the interpretation of calcium–phosphate disturbances. Combined with other still scarce data on intact FGF23 values in healthy children, we provide an useful tool for recognising the early onset of otherwise asymptomatic early stages of bone disorders in children of the population at risk, especially CKD children. Values of intact FGF23 measured in our population can serve as a reference point under clinical conditions and for other studies on the topic.

## 5 Conclusion

Our study supports the evidence that FGF23 level in paediatric population is not age or sex dependent.

## References

[1] Consortium A. Autosomal dominant hypophosphataemic rickets is associated with mutations in FGF23. Nat Genet. 2000;26(3):345–8. 10.1038/81664 11062477

[2] Gattineni J, Baum M. Regulation of phosphate transport by fibroblast growth factor 23 (FGF23): implications for disorders of phosphate metabolism. Pediatr Nephrol. 2010;25(4):591–601. 10.1007/s00467-009-1273-z PMC3151467 19669798

[3] Krajisnik T, Bjorklund P, Marsell R, Ljunggren O, Akerstrom G, Jonsson KB, et al. Fibroblast growth factor-23 regulates parathyroid hormone and 1alpha-hydroxylase expression in cultured bovine parathyroid cells. J Endocrinol. 2007;195(1):125–31. 10.1677/JOE-07-0267 17911404

[4] Shimada T, Hasegawa H, Yamazaki Y, Muto T, Hino R, Takeuchi Y, et al. FGF-23 is a potent regulator of vitamin D metabolism and phosphate homeostasis. J Bone Miner Res. 2004;19(3):429–35. 10.1359/JBMR.0301264 15040831

[5] Urakawa I, Yamazaki Y, Shimada T, Iijima K, Hasegawa H, Okawa K, et al. Klotho converts canonical FGF receptor into a specific receptor for FGF23. Nature. 2006;444(7120):770–4. 10.1038/nature05315 17086194

[6] Shimada T, Muto T, Urakawa I, Yoneya T, Yamazaki Y, Okawa K, et al. Mutant FGF-23 responsible for autosomal dominant hypophosphatemic rickets is resistant to proteolytic cleavage and causes hypophosphatemia in vivo. Endocrinology. 2002;143(8):3179–82. 10.1210/endo.143.8.8795 12130585

[7] Bacchetta J, Dubourg L, Harambat J, Ranchin B, Abou-Jaoude P, Arnaud S, et al. The influence of glomerular filtration rate and age on fibroblast growth factor 23 serum levels in pediatric chronic kidney disease. J Clin Endocrinol Metab. 2010;95(4):1741–8. 10.1210/jc.2009-1576 20157196

[8] Ziolkowska H, Okarska-Napierala M, Stelmaszczyk-Emmel A, Gorska E, Zachwieja K, Zurowska A, et al. Serum fibroblast growth factor 23 and calcium-phosphorus metabolism parameters in children with chronic kidney disease – preliminary report. Dev Period Med. 2014;18(2):194–202. 25182258

[9] Portale AA, Wolf M, Juppner H, Messinger S, Kumar J, Wesseling-Perry K, et al. Disordered FGF23 and mineral metabolism in children with CKD. Clin J Am Soc Nephrol. 2014;9(2):344–53. 10.2215/CJN.05840513 PMC3913243 24311704

[10] Desjardins L, Liabeuf S, Renard C, Lenglet A, Lemke HD, Choukroun G, et al. FGF23 is independently associated with vascular calcification but not bone mineral density in patients at various CKD stages. Osteoporos Int. 2012;23(7):2017–25. 10.1007/s00198-011-1838-0 22109743

[11] Gutierrez OM. Fibroblast growth factor 23 and disordered vitamin D metabolism in chronic kidney disease: updating the “trade-off” hypothesis. Clin J Am Soc Nephrol. 2010;5(9):1710–6. 10.2215/CJN.02640310 20507957

[12] Fischer DC, Mischek A, Wolf S, Rahn A, Salweski B, Kundt G, et al. Paediatric reference values for the c-terminal fragment of fibroblast-growth factor-23, sclerostin, bone-specific alkaline phosphatase and isoform 5b of tartrate-resistant acid phosphatase. Ann Clin Biochem. 2012;49(Pt 6):546–53. 10.1258/acb.2012.011274 22984195

[13] Shimada T, Mizutani S, Muto T, Yoneya T, Hino R, Takeda S, et al. Cloning and characterization of FGF23 as a causative factor of tumor-induced osteomalacia. Proc Natl Acad Sci USA. 2001;98(11):6500–5. 10.1073/pnas.101545198 PMC33497 11344269

[14] Jonsson KB, Zahradnik R, Larsson T, White KE, Sugimoto T, Imanishi Y, et al. Fibroblast growth factor 23 in oncogenic osteomalacia and X-linked hypophosphatemia. N Engl J Med. 2003;348(17):1656–63. 10.1056/NEJMoa020881 12711740

[15] Faul C, Amaral AP, Oskouei B, Hu MC, Sloan A, Isakova T, et al. FGF23 induces left ventricular hypertrophy. J Clin Invest. 2011;121(11):4393–408. 10.1172/JCI46122 PMC3204831 21985788

[16] Ix JH, Shlipak MG, Wassel CL, Whooley MA. Fibroblast growth factor-23 and early decrements in kidney function: the heart and soul study. Nephrol Dial Transplant. 2010;25(3):993–7. 10.1093/ndt/gfp699 PMC2902926 20037168

[17] Bergwitz C, Juppner H. Regulation of phosphate homeostasis by PTH, vitamin D, and FGF23. Annu Rev Med. 2010;61:91–104. 10.1146/annurev.med.051308.111339 PMC4777331 20059333

[18] National Kidney F. K/DOQI clinical practice guidelines for bone metabolism and disease in chronic kidney disease. Am J Kidney Dis. 2003;42(4 Suppl 3):S1–201. 14520607

[19] Gkentzi D, Efthymiadou A, Kritikou D, Chrysis D. Fibroblast growth factor 23 and Klotho serum levels in healthy children. Bone. 2014;66:8–14. 10.1016/j.bone.2014.05.012 24880094

[20] Mitchell DM, Juppner H, Burnett-Bowie SM. FGF23 is not associated with age-related changes in phosphate, but enhances renal calcium reabsorption in girls. J Clin Endocrinol Metab. 2017;102(4):1151–60. 10.1210/jc.2016-4038 PMC5460726 28323960

[21] Berndt T, Thomas LF, Craig TA, Sommer S, Li X, Bergstralh EJ, et al. Evidence for a signaling axis by which intestinal phosphate rapidly modulates renal phosphate reabsorption. Proc Natl Acad Sci U S A. 2007;104(26):11085–90. 10.1073/pnas.0704446104 PMC1891094 17566100

[22] Giral H, Caldas Y, Sutherland E, Wilson P, Breusegem S, Barry N, et al. Regulation of rat intestinal Na-dependent phosphate transporters by dietary phosphate. Am J Physiol Renal Physiol. 2009;297(5):F1466–75. 10.1152/ajprenal.00279.2009 PMC2781338 19675183

[23] Braithwaite V, Jarjou LM, Goldberg GR, Prentice A. Iron status and fibroblast growth factor-23 in Gambian children. Bone. 2012;50(6):1351–6. 10.1016/j.bone.2012.03.010 PMC3360160 22465847

[24] Braithwaite V, Prentice AM, Doherty C, Prentice A. FGF23 is correlated with iron status but not with inflammation and decreases after iron supplementation: a supplementation study. Int J Pediatr Endocrinol. 2012;2012(1):27. 10.1186/1687-9856-2012-27 PMC3523041 23098062

[25] Durham BH, Joseph F, Bailey LM, Fraser WD. The association of circulating ferritin with serum concentrations of fibroblast growth factor-23 measured by three commercial assays. Ann Clin Biochem. 2007;44(Pt 5):463–6. 10.1258/000456307781646102 17761032

[26] Fassbender WJ, Brandenburg V, Schmitz S, Sandig D, Simon SA, Windolf J, et al. Evaluation of human fibroblast growth factor 23 (FGF-23) c-terminal and intact enzyme-linked immunosorbent-assays in end-stage renal disease patients. Clin Lab. 2009;55(3–4):144–52. 19462937

[27] Devaraj S, Duncan-Staley C, Jialal I. Evaluation of a method for fibroblast growth factor-23: a novel biomarker of adverse outcomes in patients with renal disease. Metab Syndr Relat Disord. 2010;8(6):477–82. 10.1089/met.2010.0030 PMC3125567 20707671

